# The Association of Distress and Eustress with Lifestyle and Self-Perception in Adolescents Presenting to a Pediatric Emergency Department with Psychosocial Complaints

**DOI:** 10.3390/healthcare14101345

**Published:** 2026-05-14

**Authors:** Dilek Çiftci Baykal, Emrullah Konur

**Affiliations:** 1 Department of Pediatric Nursing, Faculty of Health Sciences, Van Yuzuncu Yil University, 65080 Van, Türkiye; 2 Department of Pediatric Emergency, Siirt Training and Research Hospital, 56100 Siirt, Türkiye; emrullahkonur@gmail.com

**Keywords:** adolescents, distress, eustress, lifestyle, self-perception, pediatric emergency department

## Abstract

**Background:** This study was conducted to examine the relationships between distress and eustress levels and lifestyles and self-perception in adolescents presenting to a pediatric emergency department with psychosocial complaints. **Methods:** This descriptive cross-sectional study was conducted in a single training and research hospital with 142 adolescents aged 12–17 years who presented to a pediatric emergency department with psychosocial complaints. Data were collected using an introductory information form, the Adolescent Distress–Eustress Scale, the Adolescent Lifestyle Scale, and the Self-Perception Scale for Adolescents. Pearson correlation analyses were performed to examine bivariate relationships among variables. Multiple linear regression analyses were conducted to identify variables independently associated with lifestyle and self-perception scores after controlling for relevant sociodemographic variables. Variables were entered simultaneously into the regression model. **Results:** Distress level was significantly negatively correlated with self-perception and lifestyle scores, whereas eustress level was positively correlated with both variables (*p* < 0.001). In multiple linear regression analyses, eustress, distress, and economic status were significantly and independently associated with self-perception and lifestyle scores. The models explained 45.9% and 44.8% of the variance in self-perception and lifestyle, respectively. Adolescents reporting better sleep patterns, higher academic achievement, greater self-confidence, and perceived social acceptance had higher self-perception and lifestyle scores (*p* < 0.05). **Conclusions:** These findings highlight the importance of stress perception and socioeconomic factors in understanding psychosocial well-being among adolescents presenting to pediatric emergency departments.

## 1. Introduction

Pediatric emergency departments increasingly encounter adolescents presenting with psychosocial complaints rather than life-threatening medical conditions [1,2,3,4]. Previous studies have shown that mental health problems and injuries constitute a substantial proportion of adolescents’ health service utilization [1,2,3], and approximately half of adolescent cases present for non-urgent reasons [1]. The increasing rate of adolescent presentations to pediatric emergency departments highlights the importance of understanding the clinical and psychosocial characteristics of this population [4]. In this context, a considerable proportion of adolescents seek emergency care due to psychological distress, anxiety, psychosomatic symptoms, or maladaptive help-seeking behaviors. Pediatric emergency departments therefore represent an important clinical setting for adolescent mental health.

Adolescence is defined as the transition period from childhood to adulthood, characterized by rapid physical, biochemical, mental, and social growth and maturation [5]. In the Dictionary of Psychiatry published by the American Psychiatric Association, adolescence is described as a chronological period beginning with sexual and psychosocial maturation and ending when the individual attains greater independence and social productivity [6]. Accordingly, adolescence is considered a critical developmental stage in which multidimensional biological, social, and psychological changes may render individuals more vulnerable to stress [7].

Since its emergence, stress has often been conceptualized as a reaction to challenging, threatening, or maladaptive conditions, emphasizing its negative aspect [8]. However, according to Selye, both pleasant and unpleasant experiences may produce stress [9]. Selye therefore distinguished between “eustress,” referring to positive stress experiences, and “distress,” referring to negative stress experiences associated with disappointment, failure, and humiliation [9,10]. Consistently, Hargrove et al. (2015) define eustress as a positive, constructive, and healthy response to stressful events, while Lazarus conceptualizes eustress as a constructive cognitive response associated with positive affect and a healthy physiological state [8,11]. Eustress is also linked to the Yerkes–Dodson Law, which proposes that individuals perform best under a certain level of pressure, whereas excessive and prolonged stress may reduce performance over time in an inverted U-shaped pattern [12,13,14]. When healthy lifestyle behaviors are not established and emotional development is not adequately supported during adolescence, chronic or temporary mental health problems may develop [15]. Worldwide, 10–20% of adolescents are affected by mental health problems [16]. Mental health is closely related to self-perception. The self has been defined as an internal capacity that observes, judges, evaluates, regulates, and manages behaviors, reflecting individuals’ views of themselves and how they perceive their own personality [17,18]. Within this framework, self-perception refers to adolescents’ evaluations of their own abilities and characteristics and is conceptually distinguished from related constructs such as self-esteem.

A large discrepancy between the self one aims to achieve and the self one perceives may be associated with lower self-perception and feelings of inadequacy [17,18]. Positive self-perception is linked to perceiving oneself as valuable, competent, and successful [19], and individuals with higher self-perception may adapt more effectively to different environments and life events [20]. Previous research has demonstrated associations between stress, self-perception, and lifestyle-related outcomes in adolescence [21,22,23]. However, most studies have focused primarily on the negative aspects of stress and have been conducted in school or community settings. Limited research has simultaneously examined both distress and eustress in adolescents presenting to pediatric emergency departments. Considering that pediatric emergency services often function as an important access point for adolescents with psychosocial concerns, examining stress perception within this clinical context may contribute to a more comprehensive understanding of adolescents’ lifestyle behaviors and self-perception.

Therefore, the aim of this study was to examine the associations between distress, eustress, lifestyle behaviors, and self-perception among adolescents presenting to a pediatric emergency department with psychosocial complaints. This study was conducted in a single pediatric emergency department of a training and research hospital. Adolescents presenting to pediatric emergency departments constitute a distinct clinical population characterized by increased psychosocial vulnerability, including emotional distress, risk-taking behaviors, and unmet mental health needs. Unlike community-based samples, adolescents seeking care in emergency settings often represent acute or exacerbated conditions, making this context particularly relevant for examining stress-related constructs. The pediatric emergency department serves as a critical point for early identification and intervention, especially in cases where psychosocial issues may not have been previously recognized. Therefore, investigating the relationships between distress, eustress, lifestyle, and self-perception within this clinical setting provides clinically meaningful insights that may inform targeted screening and intervention strategies.

This study was designed not only to examine relationships but also to test theoretically grounded expectations.

Based on the existing literature, the following hypotheses were formulated:

**H1.**  *Distress is negatively associated with adolescents’ self-perception and lifestyle behaviors*.

**H2.**  *Eustress is positively associated with adolescents’ self-perception and lifestyle behaviors*.

**H3.**  *Self-perception is positively associated with lifestyle behaviors*.

**H4.**  *Economic status is significantly associated with adolescents’ self-perception and lifestyle scores*.

## 2. Materials and Methods

### 2.1. Purpose and Type of Research

This research was conducted as descriptive and cross-sectional in order to examine the relationships between stress (distress and eustress) experienced in the emergency department context and lifestyles and self-perception in adolescents visiting the pediatric emergency clinic.

### 2.2. Research Population and Sample

The research population consisted of adolescents presenting with psychosocial complaints to the pediatric emergency clinic of an education and research hospital between June 1 and September 1. These complaints were identified during routine clinical assessment by pediatric emergency physicians responsible for the initial patient evaluation at admission. These assessments were conducted as part of the standard clinical workflow following triage and were based on adolescents’ presenting symptoms, including emotional distress, behavioral problems, anxiety-related complaints, and stress-related concerns. To ensure consistency in classification, all patients were evaluated using the same triage protocols and standardized clinical judgment procedures routinely applied in the unit.

Inclusion criteria included adolescents presenting to the pediatric emergency department with psychosocial complaints who agreed to participate in the study.

Exclusion criteria included adolescents with severe medical conditions requiring immediate intervention, those with cognitive impairments preventing questionnaire completion, those with previously diagnosed psychiatric disorders, those using sedative or analgesic medications, and those who declined participation. The inclusion and exclusion criteria were determined to ensure the selection of a clinically relevant and homogeneous adolescent sample presenting with psychosocial complaints, to minimize potential confounding factors that could influence stress perception, and to enhance the reliability of self-reported psychosocial measures.

A consecutive sampling approach was used. During the data collection period, all adolescents presenting to the pediatric emergency department with psychosocial complaints were screened for eligibility. Those who met the predefined inclusion criteria and agreed to participate were consecutively enrolled in the study. A total of 142 eligible adolescents agreed to participate and were included in the study. The initial sample size estimation was conducted for planning purposes; however, because multiple regression analyses were performed, an a priori power analysis was additionally conducted using G*Power (v3.1.9.7) [24]. For the multiple regression model including nine predictor variables, a medium effect size (f^2^ = 0.15), α = 0.05, and statistical power of 0.90 were assumed [25]. The analysis indicated that a minimum sample size of 141 participants was required. The present study included 142 adolescents, thereby exceeding the minimum required sample size and meeting the recommended power requirements. Furthermore, the sample size exceeded the criteria proposed by Green (1991) [26] for overall model testing (N > 50 + 8m; 142 > 50 + 72) and for testing individual predictors (N > 104 + m; 142 > 104 + 9). The participant-to-predictor ratio was approximately 16:1, which is consistent with recommendations for stable regression estimates [27].

### 2.3. Data Collection Tools

Data were collected through face-to-face administration of the questionnaires in the pediatric emergency department between 1 June and 1 September. Participants who met the inclusion criteria were informed about the study, and informed consent was obtained prior to data collection. The questionnaires were completed under the supervision of the researchers and required approximately 10–15 min. All eligible adolescents were screened consecutively upon admission to the pediatric emergency department. Questionnaires were administered only after the initial clinical evaluation and once the patient’s condition was stabilized. Participation was based entirely on voluntariness, and data collection was conducted prior to discharge. Adolescents were included only if they were in an appropriate physical and emotional condition to respond. Data collection was carried out in a quiet and private area within the emergency department, and the timing of administration was carefully arranged so as not to interfere with urgent medical care. Psychosocial complaints were identified during routine clinical assessment in the pediatric emergency department by pediatric emergency physicians responsible for initial patient evaluation. These assessments were based on standard triage and clinical evaluation procedures routinely applied in the unit. To ensure consistency in classification, all adolescents were evaluated according to the same clinical protocols and standardized triage-based clinical judgment based on presenting complaints.

Research data were obtained using the Demographic Information Form, the Adolescent Distress–Eustress Scale (ADES), the Adolescent Lifestyle Scale (ALS), and the Self-Perception Scale for Adolescents (ASES).

Adolescent Information Form: Consists of 18 questions covering adolescents’ sociodemographic characteristics, lifestyle, and school-related information [28,29,30].

Adolescent Distress–Eustress Scale (ADES): Developed by Branson et al. to comprehensively measure both distress and constructive (positive) aspects of stress responses in adolescents. The Turkish version was measured by Akgün and colleagues [14,31]. The Distress–Eustress Scale for Adolescents is a 5-point Likert-type scale consisting of ten items, each rated on a scale of 0–4 (0 = none, 1 = very little, 2 = moderate, 3 = much, 4 = a lot). The scale consists of two subscales: “Distress” and “Eustress (Constructive-Positive Stress)”. When calculating the scale score, each item is evaluated based on two subscales: the ADES -Distress total (2, 5, 6, 7, 10) and the ADES-Eustress total (1, 3, 4, 8, 9). Scores obtained from the Distress and Eustress dimensions range from 0 to 20. A total score cannot be obtained from the scale. High scores obtained from the Distress dimension indicate that stress is perceived as an experience that causes tension and distress; high scores obtained from the eustress dimension indicate that stress is perceived as constructive and motivating. The Cronbach’s alpha value of the scale was found to be 0.81 for the distress subscale and 0.84 for the eustress subscale. In this study, the Cronbach’s alpha was 0.71 for the distress subscale and 0.72 for the eustress subscale.

Adolescent Lifestyle Scale (ALS): This is the adolescent version of the Healthy Lifestyle Scale II, developed based on the Health Promotion Model. The scale was developed by Hendricks in 2006, and its validity and reliability were tested for the Turkish version by Ardıç [28,29]. The scale enables the determination of healthy lifestyle behaviors in early, middle, and late adolescents. The scale consists of 40 items and seven independent subgroups. All items of the Adolescent Lifestyle Scale are positive. The scale requires a 4-point Likert-type response for each item. A score of 1 is given for “Never,” 2 for “Sometimes,” 3 for “Often,” and 4 for “Always.” The maximum score obtainable from the scale is 160, and the minimum score is 40. The scale has no cutoff point; as the score increases, the level of positive health behavior increases. The Cronbach’s alpha value of the scale was found to be 0.87. In this study, the Cronbach’s alpha value of the scale was found to be 0.85.

*Self-Perception Scale for Adolescents (ASES):* The Self-Perception Scale for Adolescents is a 25-item measure. Each item is rated on a 5-point Likert scale. Higher scores indicate higher levels of self-perception. The scale has a multidimensional structure. The Cronbach’s alpha value of the scale was reported as 0.91 in previous studies [30]. In the present study, the Cronbach’s alpha coefficient of the ASES was found to be 0.92, indicating high internal consistency.

### 2.4. Data Evaluation

The data analysis was performed using IBM SPSS Statistics (Windows, version 26.0.) for Windows, version 26.0 (IBM Corp., Armonk, NY, USA). Descriptive statistics are presented as counts, percentages, means, and standard deviations. The assumption of normal distribution was evaluated by considering skewness and kurtosis values. In intergroup comparisons, the independent samples *t*-test and one-way analysis of variance (ANOVA) were used when assumptions were met, and the Mann–Whitney U and Kruskal–Wallis H tests were used when assumptions were not met. Bonferroni correction was applied in multiple comparisons where significant differences were detected. The relationships between variables were examined using Pearson correlation analysis. The reliability of the scales was assessed using Cronbach’s alpha coefficient. Multiple linear regression analysis was performed to examine the variables associated with self-perception and lifestyle scores; prior to analysis, the assumptions of normality, multicollinearity, and homogeneity of variances were checked. Multicollinearity among the independent variables was assessed using tolerance and Variance Inflation Factor (VIF) statistics. The tolerance values ranged between 0.524 and 0.929, while VIF values ranged between 1.077 and 1.908. Since all VIF values were below the recommended threshold of 5 and tolerance values were above 0.20, it was concluded that there was no evidence of multicollinearity in the regression model [32,33]. Effect sizes were calculated using Cohen’s d for independent group comparisons and interpreted as small (0.20), medium (0.50), and large (0.80). For ANOVA analyses, eta-squared values were reported. For regression models, adjusted R^2^ values were used to evaluate model fit.

## 3. Results

The sociodemographic and anthropometric characteristics of the participants are presented in Table 1.

The sociodemographic characteristics of the participants are presented in Table 1. Of the participants, 40.8% were female and 59.2% were male. A total of 57.0% were attending middle school, and 43.0% were attending high school. Academic performance was reported as average by 59.2% of the participants, followed by good (33.1%) and poor (7.7%). In terms of economic status, 54.2% reported that their income was equal to their expenses, 30.3% reported income higher than expenses, and 15.5% reported income lower than expenses. The majority lived in nuclear families (72.5%), followed by extended families (23.2%) and separated families (4.2%).

The mean age of the participants was 14.6 ± 1.69 years, the mean number of siblings was 3.99 ± 1.91, and the mean body mass index (BMI) was 20.2 ± 4.82 (range: 12.7–50.3).

The health-related and lifestyle characteristics of the participants are presented in Table 2.

The health-related and lifestyle characteristics of the participants are presented in Table 2. Sleeping 5–8 h per day was reported by 53.5% of the participants, and 59.9% reported average sleep quality. Technology use was reported as moderate by 50.0% of the participants. A total of 98.6% of the participants did not work outside of school. Regarding stress management, 46.5% reported listening to music, 29.6% reported exercising, 16.9% reported avoiding stressful environments, and 7.0% reported seeking support. A total of 83.8% reported being physically and mentally fit, 69.7% described themselves as self-confident, and 78.2% reported being socially accepted.

Descriptive statistics and correlations of the study variables are presented in Table 3.

The mean ALS score was 105.0 ± 15.5, and the mean ASES score was 92.3 ± 16.8. The mean scores for ADES-Distress and ADES-Eustress were 10.7 ± 5.3 and 12.0 ± 4.62, respectively. Cronbach’s alpha values ranged from 0.71 to 0.92.

Pearson correlation analysis results are presented in Table 4.

Pearson correlations between number of siblings and outcome variables are presented in Table 5.

ASES (r = −0.258, *p* = 0.002) and ALS (r = −0.256, *p* = 0.002) were negatively correlated with the number of siblings.

Comparisons of ASES and ALS scores according to participant characteristics and effect sizes are presented in Table 6.

Model summary results of the multiple linear regression analyses are presented in Table 7.

Both regression models were statistically significant (Model 1: F(9.132) = 12.5, *p* < 0.001; Model 2: F(9.132) = 11.9, *p* < 0.001). For Model 1 (ASES), R = 0.678, R^2^ = 0.459, and Adjusted R^2^ = 0.422. For Model 2 (ALS), R = 0.669, R^2^ = 0.448, and Adjusted R^2^ = 0.410.

Regression coefficients for predictors are presented in Table 8.

For Model 1 (ASES), eustress (B = 1.32, SE = 0.32, β = 0.362, *p* < 0.001), distress (B = −0.73, SE = 0.26, β = −0.230, *p* = 0.006), and economic status (B = 4.24, SE = 1.75, β = 0.161, *p* = 0.016) were statistically significant predictors. Other variables were not statistically significant (*p* > 0.05).

For Model 2 (ALS), distress (B = −0.86, SE = 0.25, β = −0.294, *p* = 0.001), eustress (B = 0.97, SE = 0.30, β = 0.289, *p* = 0.002), and economic status (B = 4.51, SE = 1.62, β = 0.181, *p* = 0.006) were statistically significant predictors. Other variables were not statistically significant (*p* > 0.05).

## 4. Discussion

The findings of this study were consistent with the proposed hypotheses. Distress was negatively associated with self-perception and lifestyle behaviors, whereas eustress was positively associated with these variables. In addition, self-perception was positively associated with lifestyle behaviors. Economic status and academic achievement were significantly associated with both self-perception and lifestyle scores. These findings are consistent with previous research indicating that both negative and positive dimensions of stress are associated with adolescents’ psychosocial outcomes.

This study examined the relationships between eustress and distress levels of adolescents attending a pediatric emergency department and their self-perception and lifestyle characteristics. The results indicate that stress perception in this context was associated with adolescents’ psychosocial characteristics. Distress was negatively associated with self-perception and lifestyle scores, whereas eustress was positively associated with these outcomes. Correlation and regression analyses showed that distress was negatively associated with both outcomes. Although distress and eustress were negatively correlated, they represent distinct dimensions of stress perception and were therefore included simultaneously in the regression model to examine their independent statistical associations. These findings are in line with Lazarus and Folkman’s cognitive appraisal model of stress [8], which suggests that stress perceived as threatening is associated with adverse psychosocial outcomes.

In the present study, adolescents were evaluated in a pediatric emergency department despite not necessarily presenting with severe medical conditions. This finding may reflect the fact that presentations to the emergency department also involve psychosocial concerns. In this context, stress perception in the emergency setting may be associated with broader psychosocial characteristics. Taken together, these findings are consistent with previous theoretical and empirical work indicating that both distress and eustress are associated with adolescents’ psychosocial outcomes. Previous literature also reports that adolescents with lower self-perception tend to report higher perceived stress levels. This finding is consistent with previous research showing that adolescents with lower self-perception tend to report higher perceived stress and poorer mental well-being [21,34]. Longitudinal studies have also indicated that stress levels are related to both short- and long-term lifestyle patterns [35]. In addition, higher levels of eustress were associated with higher self-perception and healthier lifestyle scores. Unlike traditional stress-focused models, this finding indicates that positively perceived stress is associated with positive developmental outcomes.

However, given the cross-sectional design of the study, these findings should be interpreted as statistical associations rather than causal relationships. This pattern is in line with theoretical approaches such as the Yerkes–Dodson law and positive stress perspectives [12]. The study’s findings indicate a positive association between eustress and self-perception. This pattern indicates that adolescents may not experience stress solely as a negative condition, and certain forms of stress are perceived as constructive during development. Previous studies have also reported that positive stress experiences are associated with self-efficacy, motivation, problem-solving capacity, and social coping skills [11,36]. These findings are consistent with existing literature highlighting the role of eustress as a component of stress perception in adolescents within a clinical emergency context.

In our study, higher eustress scores were associated with higher self-perception and lifestyle scores. Additionally, self-perception and healthy lifestyle behaviors were positively related. Maintaining healthy lifestyle behaviors—such as regular sleep, balanced nutrition, social participation, and emotional regulation—was associated with higher self-perception, while higher self-perception was associated with more effective coping and the continuation of health-promoting behaviors [16,37]. This two-way relationship indicates that self-perception and lifestyle are closely related during adolescence. The literature indicates that adolescents who do not develop healthy lifestyle habits tend to have lower self-perception and may be more sensitive to stress [16,37]. Similarly, González Moreno and Molero Jurado (2023) [22] reported a negative relationship between healthy lifestyle behaviors and stress in adolescents, and a positive relationship between healthy lifestyle and self-perception. Haapala et al. (2025) [38] also reported that healthy lifestyle habits from childhood to adolescence are associated with lower perceived stress and depression during adolescence. In our study, the decline in lifestyle scores accompanying increased stress is consistent with these findings. Carter (2018) [23] reported longitudinal associations between stress, self-perception, and later physical activity and sedentary behavior patterns. In addition, the positive association observed between eustress and self-perception in this study reflects statistical associations rather than causal effects.

Eustress, arising from the positive perception of stress, has been described as an adaptive psychological response in the literature [31,39,40]. Previous research has shown that adolescents with higher levels of eustress tend to report better psychological well-being, self-efficacy, and healthier behaviors [41]. In our study higher eustress scores were associated with higher self-perception and lifestyle scores, which is consistent with these findings. However, given the cross-sectional design, these results reflect statistical associations rather than causal relationships. The findings show associations between stress perception, self-perception, and lifestyle behaviors during adolescence. Higher distress levels were associated with lower self-perception and less favorable lifestyle patterns, whereas higher eustress was associated with more positive psychosocial indicators.

Economic status was found to be significantly associated with both self-perception and lifestyle scores. Although its standardized coefficient was lower than those of distress and eustress, it remained statistically significant in the regression model. Socioeconomic conditions were associated with adolescents’ psychosocial outcomes. However, given the cross-sectional nature of the study, these findings reflect statistical associations rather than directional effects. Higher perceived economic status was statistically associated with more favorable stress perception patterns and higher lifestyle scores in this sample. Previous studies indicate that adolescents’ emergency department visits are related to socioeconomic variables. The decrease in self-perception and lifestyle scores observed with a higher number of siblings is reported in relation to shared family resources, as previous literature suggests that family size can influence adolescents’ psychosocial outcomes [1,42]. Palabıyıkoğlu et al. (1989) [43] similarly reported that families with many children experienced marital conflict, which negatively affected adolescents’ level of self-acceptance.

Quality sleep enables individuals to feel refreshed and ready for daily activities, and includes both quantitative aspects such as duration and awakenings, and qualitative aspects such as depth and restorative value of sleep [44]. In our study, although no significant difference was found based on sleep duration, adolescents who perceived their sleep quality as poor had lower self-perception and lifestyle scores. Dahl and Lewin emphasize that biological clock changes and environmental demands during adolescence lead to irregular sleep–wake cycles, increased stress sensitivity, and impaired emotional regulation and psychosocial adjustment [45]. Similarly, studies indicate that sleep problems are common among adolescents and are associated with emotional functioning, lower self-perception, and fewer health-promoting lifestyle behaviors [46]. Our findings showed that low academic achievement was significantly associated with lower self-perception and lifestyle scores. Hughes et al. (2015) [47] demonstrated that changes in school belonging during secondary education are related to academic achievement, with declines in belonging corresponding to lower achievement levels. The relationship identified in this study between academic achievement, self-perception, and lifestyle indicates an association between school experience and adolescents’ psychosocial adjustment.

The literature describes self-perception as a central psychological construct influencing goal orientation and academic behavior. Studies among university students indicate that low self-perception is associated with maladaptive achievement goals and defensive academic behaviors [48]. In our study, adolescents with low academic achievement had lower self-perception scores. The interest and support received from the environment were associated with both perceived social support and self-perception. Previous studies report that family support is associated with higher academic achievement and that peer support is related to more effective coping strategies and better school performance [49,50,51]. Similarly, Özkan (1987) [52] found that adolescents with higher self-perception reported higher academic achievement. In this study, social acceptance was identified as one of the variables most strongly associated with self-perception and lifestyle scores. Considering the importance of peer relationships during adolescence, social acceptance was associated with self-perception and daily life behaviors. The literature reports that social withdrawal and shyness are significantly associated with self-perception and psychosocial adjustment during childhood and adolescence. Rubin and colleagues emphasize that socially withdrawn individuals tend to perceive themselves as more socially inadequate, report lower self-perception, and experience greater difficulties in peer relationships. It has been noted that, particularly as peer relationships gain importance during adolescence, the effects of shyness and social withdrawal on self-perception and daily functioning may become more pronounced. In this context, our findings indicate that adolescents with low self-confidence and shy traits had lower self-perception and lifestyle scores, consistent with the existing literature [53].

The literature indicates that self-perception is positively associated with social functioning, with individuals who report higher self-perception generally feeling more comfortable and secure in social settings. Conversely, lower self-perception has been associated with social anxiety and shyness, which may be accompanied by insecurity and social withdrawal in adolescents’ interpersonal relationships. Studies conducted across different cultures and samples have reported an inverse relationship between self-perception and social anxiety [54,55]. In this context, the finding in our study that adolescents with low self-confidence were more shy is in line with the existing literature. The results of this research suggest that psychosocial well-being in adolescence is a multidimensional construct that cannot be explained by a single variable. Environmental and relational factors such as academic achievement, perceived sleep patterns, social acceptance, and number of siblings may interact with perceived stress and economic conditions in relation to self-perception and lifestyle patterns. Acceptance in social relationships and a positive perception of stress were found to be significantly associated with adolescents’ psychosocial resilience. Accordingly, the findings indicate that adolescents’ experience of stress is not one-dimensional; rather, the way stress is perceived is significantly associated with self-perception and lifestyle patterns. Higher distress levels were associated with lower self-perception and less favorable lifestyle behaviors, whereas positive stress experiences were associated with greater self-efficacy, psychological resilience, and adaptive lifestyle patterns during adolescence. In this context, psychosocial approaches aimed at adolescents may benefit from prioritizing protective interventions that support constructive stress perception rather than focusing solely on the elimination of stress.

These findings have important implications for pediatric nursing practice. Adolescents presenting to pediatric emergency settings often undergo primarily medical evaluation, while psychosocial stress perception may remain under-recognized. The present results indicate that distress was associated with lower self-perception and less favorable lifestyle behaviors, whereas eustress was positively associated with psychological indicators. Therefore, pediatric nurses working in emergency departments are in a key position to assess stress perception, identify adolescents at psychosocial risk, and initiate early supportive interventions or referrals when necessary. Integrating brief psychosocial screening into routine nursing assessment may contribute to more holistic and preventive care for adolescents.

### Strengths and Limitations

This study has several limitations. First, due to its cross-sectional design, causal inferences cannot be drawn and the directionality of the associations between stress perception, self-perception, and lifestyle cannot be determined; therefore, the findings should be interpreted as statistical associations rather than causal relationships. Second, self-reported data may be subject to response bias, as the data rely on participants’ self-reports.

The main strength of this study is that it provides a more comprehensive assessment of stress perception in adolescents by addressing both eustress and distress simultaneously. The use of validated and reliable measurement tools and the reporting of effect sizes enhance the interpretability of the findings. Furthermore, the examination of multiple psychosocial variables such as school success, perceived sleep patterns, social relationship status, and number of siblings allows for a multidimensional assessment of factors related to self-perception and lifestyle.

Data were collected during the summer holiday period (June–September). Academic stress levels are known to fluctuate across the school year, and the timing of data collection may have influenced the observed stress levels. Therefore, the findings may not fully reflect stress patterns during other academic periods, which may limit the generalizability of the results across the entire year. In addition, the subjective assessment of sleep patterns and the measurement of economic status using self-reported indicators may further limit the generalizability of the findings. Finally, the fact that the sample was selected from a single region may restrict the generalization of the results to different sociocultural contexts.

Additionally, as the sample consisted of adolescents presenting to a pediatric emergency department with psychosocial complaints, the findings may not fully reflect the typical psychosocial functioning of adolescents in the general population. Adolescents presenting in this clinical context may differ from the general population in terms of stress perception and psychosocial characteristics.

## 5. Conclusions

The findings of this study indicate that adolescents’ perceptions of stress in the emergency department context were significantly associated with their self-perception and lifestyle behaviors. Distress was negatively associated with these outcomes, whereas eustress showed a positive association.

These findings suggest that stress may not be uniformly harmful; rather, the way stress is perceived appears to be closely related to self-perception and lifestyle patterns. Psychosocial approaches for adolescents may focus on both reducing distress and promoting more constructive stress perceptions. However, longitudinal studies are needed to clarify the directionality of these associations.

## Figures and Tables

**Table 1:** Sociodemographic characteristics of the participants (*n* = 142).

Variables	Categories	*n*	(%)
Gender	Female	58	40.8
	Male	84	59.2
Educational status	Middle school	81	57
	High school	61	43
School academic performance	Poor	11	7.7
	Average	84	59.2
	Good	47	33.1
Economic situation	Income less than expenses	22	15.5
	Income equals expenses	77	54.2
	Income more than expenses	43	30.3
Family type	Extended family	33	23.2
	Nuclear family	103	72.5
	Broken family (parents separated)	6	4.2

*n*: Number (frequency); %: Percentage.

**Table 2:** Health-related lifestyle variables (*n* = 142).

Variables	Categories	*n*	(%)
Family history of psychiatric illness	Yes	4	2.8
	No	138	97.2
Average sleep duration (hours)	0–4	10	7
	5–8	76	53.5
	9–12	56	39.4
Sleep pattern assessment	Good	29	20.4
	Average	85	59.9
	Bad	28	19.7
Technology use status	Low	24	16.9
	Average	71	50
	High	47	33.1
Working outside of school	Yes	2	1.4
	No	140	98.6
Stress management techniques	Exercising	42	29.6
	Listening to music	66	46.5
	Avoiding stressful environments	24	16.9
	Getting support	10	7
Physical and mental fitness	Yes	119	83.8
	No	23	16.2
Self-definition	Confident	99	69.7
	Shy	43	30.3
Social relation status	Accepted	111	78.2
	Rejected	31	21.8

*n*: Number, %: Percentage.

**Table 3:** Descriptive statistics of the study variables.

	N	Mean	SD	Min	Max	Range	Cronbach’s Alpha
ALS	142	105	15.5	68	146	40–160	0.85
ASES	142	92.3	16.8	30	125	25–125	0.92
ADES-Stress	142	10.7	5.3	2	20	0–20	0.71
ADES-Eustress	142	12	4.62	2	20	0–20	0.72

N: Number; SD: Standard deviation; Min: Minimum; Max: Maximum. Range indicates the theoretical score range of each scales. ALS: Adolescent Lifestyle Scale (5-point Likert type scale with 40 questions, with values between 40 and 160 points). ASES: Self-Perception Scale for Adolescents (5-point Likert type scale with 25 questions, with values between 25 and 125 points). ADES: Adolescent Distress–Eustress Scale (the scale evaluated according to sub-dimensions is of 4-point Likert type (0–4), with values between 0 and 20 points).

**Table 4:** Pearson correlations among study variables.

	ALS	ASES	ADES-Stress	ADES-Eustress
ALS	1	r = 0.644	r = −0.548	r = 0.552
		**	**	**
		r^2^ = 0.41	r^2^ = 0.30	r^2^ = 0.30
ASES		1	r = −0.532	r = 0.593
			**	**
			r^2^ = 0.28	r^2^ = 0.35
ADES-Stress			1	r = −0.556
				**
				r^2^ = 0.31

r: Pearson correlation coefficient; r^2^: coefficient of determination (shared variance); ** *p* < 0.001.

**Table 5:** Pearson correlations between number of siblings and outcome variables (N = 142).

**Variable**	**r**	***p***
ASES	−0.258	0.002
ALS	−0.256	0.002

r: Pearson correlation coefficient; *p* values are two-tailed; N = 142.

**Table 6:** Comparison of ASES and ALS scores according to variables and effect size.

		ASES			ALS		
Variables	Categories	Mean ± SD	Test Statistic		Mean ± SD	Test Statistic	
Gender	Female	94.4 ± 14.9	*t*: 1.277		105.5 ± 15.5	*t*: 0.292	
	Male	90.8 ± 18	*p*: 0.204		104.7 ± 15.6	*p*: 0.771	
Education level	Middle school	91.7 ± 17	*t*: −0.431		104.5 ± 14.8	*t*: −0.421	
	High school	93 ± 16.8	*p*: 0.667		105.6 ± 16.5	*p*: 0.675	
School academic performance	Poor ^(1)^	76.7 ± 16.9	F: 7.655	η^2^: 0.10	92.1 ± 11.7	F: 4.775	η^2^: 0.06
	Moderate ^(2)^	91.4 ± 16.7	*p*: 0.001 *	^1 < 2^ **	105.2 ± 14.9	*p*: 0.010 *	^1 < 2^ **
	Good ^(2)^	97.5 ± 14.7			107.7 ± 16.1		
Economic status	Income less than expenses ^(1)^	85.1 ± 19.6	F: 5.136	η^2^: 0.07	97.7 ± 14.3	F: 6.086	η^2^: 0.08
	Income equal to expenses ^(2)^	95.1 ± 15.7	*p*: 0.007 *	^1 < 2^ **	107 ± 15.6	*p*: 0.003 *	^1 < 2^ **
	Income greater than expenses ^(2)^	98.9 ± 15			111.5 ± 13.6		
Family type	Extended family	91.2 ± 14.8	F: 0.189		104.3 ± 13.9	F: 0.644	
	Nuclear family	92.7 ± 17.7	*p*: 0.828		105.6 ± 16.2	*p*: 0.527	
	Divorced family	89.5 ± 12.7			98.5 ± 12.3		
Psychiatric illness in family	Yes	93.3 ± 12.6	Z: −0.068		109.3 ± 15.6	Z: −0.574	
	No	92.2 ± 17	*p*: 0.946		104.9 ± 15.5	*p*: 0.566	
Sleep duration (hours)	0–4	87.6 ± 18.8	F: 0.718		99.5 ± 17.8	F: 0.792	
	5–8	91.6 ± 17.5	*p*: 0.489		104.9 ± 15.2	*p*: 0.455	
	9 or more	93.9 ± 15.7			106.2 ± 15.6		
Sleep quality evaluation	Good ^(1)^	97 ± 13.8	F: 5.340	η^2^: 0.07	107.8 ± 14.9	F: 3.504	η^2^: 0.05
	Moderate ^(1)^	92.3 ± 16.1	*p*: 0.006 *	^1 > 2^ **	105.6 ± 15.1	*p*: 0.033 *	^1 > 2^ **
	Poor ^(2)^	83 ± 21.2			97.5 ± 16.3		
Technology use	Low	97.3 ± 15.6	F: 1.505		109.2 ± 15.9	F: 1.837	
	Moderate	92 ± 16.7	*p*: 0.226		105.6 ± 16.4	*p*: 0.163	
	High	90 ± 17.5			102 ± 13.4		
Employment outside school	Yes	75 ± 8.5	Z: 1.628		97.5 ± 20,5	Z: 0.701	
	No	92.5 ± 16.8	*p*: 0.115		105.1 ± 15.5	*p*: 0.509	
Stress coping method	Doing sports	94.1 ± 16.4	KW: 2.344		108.8 ± 15.4	F: 2.105	
	Listening to music	92.9 ± 16.7	*p*: 0.504		104.9 ± 15.7	*p*: 0.102	
	Avoiding stress	87.2 ± 18.2			99 ± 15.1		
	Seeking support	92.3 ± 16.4			103.9 ± 12.9		
Physical and mental fitness	Yes	93.8 ± 15.5	*t*: 2.575	d: 0.41	106.6 ± 15.4	*t*: 2.850	d: 0.46
	No	84.1 ± 21	*p*: 0.011 *		96.8 ± 13.8	*p*: 0.005 *	
Self-definition	Self-confident	95 ± 15.2	*t*: 3.042	d: 0.51	108.4 ± 14.5	*t*: 4.134	d: 0.70
	Shy	85.9 ± 18.7	*p*: 0.003 *		97.3 ± 15	*p* < 0.001 *	
Social relationship status	Accepted	95.8 ± 14.7	*t*: 5.163	d: 0.87	108 ± 14.8	*t*: 4.730	d: 0.81
	Rejected	79.5 ± 18.1	*p* < 0.001 *		94.2 ± 13	*p* < 0.001 *	
Age			r: −0.120			r: −0.022	
			*p*: 0.155			*p*: 0.791	
BMI			r: 0.068			r: −0.025	
			*p*: 0.424			*p*: 0.772	
Number of siblings			r: −0.256	r^2^: 0.06		r: −0.258	r^2^: 0.07
			*p*: 0.002 *			*p*: 0.002 *	

SD: Standard deviation; *t*: independent samples *t*-test; F: one-way ANOVA; Z: Mann–Whitney U test; KW: Kruskal–Wallis test; η^2^: eta squared effect size; d: Cohen’s d effect size; r: Pearson correlation coefficient; * *p* < 0.05; **. *p* < 0.01; ^(1)^, ^(2)^: Post hoc group comparisons (Bonferroni-adjusted).

**Table 7:** Model summary of multiple linear regression analyses predicting ASES and ALS scores.

Model	R	R Square	Adjusted R Square	Std. Error of the Estimate	F	df1	df2	Significance
1	0.678 ^a^	0.459	0.422	12.8	12.5	9	132	<0.001
2	0.669 ^a^	0.448	0.410	11.9	11.9	9	132	<0.001

Model 1 dependent variable: ASES. Model 2 dependent variable: ALS. ^a^ Predictors: Predictors: constant, eustress, distress, sleep pattern assessment, physical and mental fitness, economic status, academic performance, self-definition, number of siblings, social relationship status.

**Table 8:** Regression coefficients for predictors of ASES and ALS scores.

	B	Standard Error	β	*p*
Model 1				
Constant	91.6	11.8		<0.001
Eustress	1.32	0.32	0.362	<0.001
Stress	−0.73	0.26	−0.23	0.006 *
Economic status	4.24	1.75	0.161	0.016 *
School academic performance	2.94	1.96	0.102	0.136
Social relation status	−3.62	3.36	−0.089	0.283
Physical and mental fitness	−2.34	3.03	−0.051	0.44
Sleep pattern assessment	−0.56	1.83	−0.022	0.761
Number of Siblings	0.11	0.63	0.013	0.861
Self-definition	−0.09	2.67	−0.003	0.973
Model 2				
Constant	123.7	10.9		<0.001
Stress	−0.86	0.25	−0.294	0.001 *
Eustress	0.97	0.3	0.289	0.002 *
Economic status	4.51	1.62	0.186	0.006 *
Self-definition	−4.14	2.49	−0.123	0.098
Physical and mental fitness	−3.53	2.82	−0.084	0.212
Social relation status	−2.1	3.13	−0.056	0.503
Sleep pattern assessment	−0.9	1.7	−0.038	0.6
School academic performance	0.57	1.82	0.021	0.756
Number of Sibling	0.13	0.59	0.016	0.827

Model 1 dependent variable: ASES; Model 2 dependent variable: ALS. B: Unstandardized coefficient; SE: Standard error; β: Standardized coefficient; * *p* < 0.05.

## Data Availability

The data presented in this study are available on request from the corresponding author (the data are not publicly available due to privacy restrictions).

## References

[1] Ziv A., Boulet J.R., Slap G.B. (1998). Emergency department utilization by adolescents in the United States. Pediatrics.

[2] Melzer-Lange M., Lye P.S. (1996). Adolescent health care in a pediatric emergency department. Ann. Emerg. Med..

[3] Van Amstel L.L., Lafleur D.L., Blake K. (2004). Raising our HEADSS: Adolescent psychosocial documentation in the emergency department. Acad. Emerg. Med..

[4] Wilson K.M., Klein J.D. (2000). Adolescents who use the emergency department as their usual source of care. Arch. Pediatr. Adolesc. Med..

[5] Ball J.W., Bindler R.C. (2006). Child Health Nursing: Partnering with Children and Families.

[6] Eriş B. (2007). The Application of the Relational Developmental Approach Model in Enhancing Parenting Competence in Mothers. Doctoral Dissertation.

[7] Cicchetti D., Rogosch F.A. (2002). A developmental psychopathology perspective on adolescence. J. Consult. Clin. Psychol..

[8] Lazarus R.S., Folkman S. (1984). Stress, Appraisal, and Coping.

[9] Selye H., Serban G. (1976). Stress without distress. Psychopathology of Human Adaptation.

[10] Salgırlı Demirbaş Y. (2015). The effects of stress on behavior in dogs. Mehmet Akif Ersoy Univ. Health Sci. J..

[11] Hargrove M.B., Becker W.S., Hargrove D.F. (2015). The HRD eustress model: Generating positive stress with challenging work. Hum. Resour. Dev. Rev..

[12] Yerkes R.M., Dodson J.D. (1908). The relation of strength of stimulus to rapidity of habit formation. J. Comp. Neurol. Psychol..

[13] Venkatesh B., Ram N. (2015). Eustress: A unique dimension to stress management. Voice Res..

[14] Akgün N., Sevim E., Ekşi F., Ekşi H. (2021). Turkish adaptation of the Distress–Eustress Scale for adolescents. Turk. J. Child Adolesc. Ment. Health.

[15] Yılmaz M., Çelebioğlu A. (2019). Protection and development of mental health in adolescents. Adolescent Health and Nursing Approaches.

[16] Skundberg-Klethagen H., Moen Ø. (2017). Mental health work in school health services and school nurses’ involvement and attitudes. J. Clin. Nurs..

[17] Öz F. (2004). Basic Concepts in Health.

[18] Budak S. (2005). Dictionary of Psychology.

[19] Kılıççı Y. (1992). Mental Health at School.

[20] Tutar H., Altınöz M., Çakıroğlu D. (2009). Evaluation of employees’ self-perceptions in terms of individual characteristics. Selçuk Univ. J. Soc. Sci. Inst..

[21] Yıldız M., Baytemir K., Demirtaş A. (2018). Irrational beliefs and perceived stress in adolescents: The role of self-esteem. J. Educ. Sci. Psychol..

[22] González Moreno A., Molero Jurado M.D.M. (2023). Healthy lifestyle in adolescence: Associations with stress, self-esteem and the roles of school violence. Healthcare.

[23] Carter J.S. (2018). Stress and self-esteem in adolescence predict physical activity and sedentary behavior in adulthood. Ment. Health Phys. Act..

[24] Faul F., Erdfelder E., Buchner A., Lang A.G. (2009). Statistical power analyses using G* Power 3.1: Tests for correlation and regression analyses. Behav. Res. Methods.

[25] Cohen J. (1977). Statistical Power Analysis for the Behavioral Sciences.

[26] Green S.B. (1991). How many subjects does it take to do a regression analysis. Multivar. Behav. Res..

[27] Stevens J. (2002). Applied Multivariate Statistics for the Social Sciences.

[28] Hendricks C.S., Murdaugh C., Pender N.J. (2006). The adolescent lifestyle profile: Development and psychometric characteristics. J. Natl. Black Nurses Assoc..

[29] Ardıç A., Esin M.N. (2015). The adolescent lifestyle profile scale: Reliability and validity of the Turkish version. J. Nurs. Res..

[30] Şahin E., Ersanlı K. (2021). Development of a self-perception scale for adolescents. OPUS Int. J. Soc. Res..

[31] Branson V., Dry M.J., Palmer E., Turnbull D. (2019). The adolescent distress–eustress scale: Development and validation. SAGE Open.

[32] Hair J.F., Black W.C., Babin B.J., Anderson R.E. (2019). Multivariate Data Analysis.

[33] Menard S. (2002). Applied Logistic Regression Analysis.

[34] Gökhan Ü., Ceylan R. (2025). Demografik ve Sosyal Faktörlerin Adölesanların Sağlıklı Yaşam Biçimi Davranışlarına Etkisi. Int. J. Health Exerc. Sport Sci..

[35] Carlén K., Suominen S., Augustine L. (2023). The association between adolescents’ self-esteem and perceived mental well-being in Sweden: A four-year follow-up. BMC Psychol..

[36] Steinberg L. (2013). Adolescence.

[37] Kaya M. (1997). The role of parental attitudes in the development of a child’s personality and self-concept. J. Fac. Theol. Ondokuz Mayıs Univ..

[38] Haapala E.A., Leppänen M.H., Kosola S., Appelqvist-Schmidlechner K., Kraav S.L., Jussila J.J., Lakka T.A. (2025). Childhood lifestyle behaviors and mental health symptoms in adolescence. JAMA Netw. Open.

[39] Le Fevre M., Matheny J., Kolt G.S. (2003). Eustress, distress, and interpretation in occupational stress. J. Manag. Psychol..

[40] Shen Y., Wang S., Chen J., Wu J. (2020). Development and validation of a Chinese version of the eustress–distress psychological response scale. Soc. Behav. Personal..

[41] Saini R., Arora A., Joshi H., Gaurav A.K. (2024). Exploring the link between eustress and adolescent health in India. J. Educ. Health Promot..

[42] Dogan H., Adıgüzel L., Uysal E., Sarıkaya S., Ozucelik D.N., Okuturlar Y., Sisek C. (2016). Differences between adolescent and adult cases of suicidal drug intoxication. Med. J. Bakirkoy.

[43] Palabıyıkoğlu R., Evrengöl A., Atakurt Y. The relationship between self-acceptance levels and parental attitudes in adolescents with neurotic symptoms. Proceedings of the XXV National Congress of Psychiatry and Neurological Sciences.

[44] İyigün G., Angın E., Kırmızıgil B., Öksüz S., Özdil A., Malkoç M. (2017). The relationship between sleep quality and mental health, physical health, and quality of life in university students. J. Exerc. Ther. Rehabil..

[45] Dahl R.E., Lewin D.S. (2002). Pathways to adolescent health: Sleep regulation and behavior. J. Adolesc. Health.

[46] Short M.A., Gradisar M., Gill J., Camfferman D. (2013). Identifying adolescent sleep problems. PLoS ONE.

[47] Hughes J.N., Im M.H., Allee P.J. (2015). Effect of school belonging trajectories in grades 6–8 on achievement. J. Sch. Psychol..

[48] Ferradás M.D.M., Freire C., Núñez J.C., Regueiro B. (2019). Associations between profiles of self-esteem and achievement goals and the protection of self-worth. Int. J. Environ. Res. Public Health.

[49] Şahin D., Güvenç G.B. (1996). Ergenlerde aile algısı ve benlik algısı. Turk. J. Psychol..

[50] Kapıkıran Ş., Özgüngör S. (2009). Ergenlerin sosyal destek düzeylerinin akademik başarı ve güdülenme ile ilişkileri. Çocuk Ve Genç. Ruh Sağlığı Derg..

[51] Cutrona C.E., Cole V., Colangelo N., Assouline S.G., Russell D.W. (1994). Perceived parental social support and academic achievement: An attachment theory perspective. J. Personal. Soc. Psychol..

[52] Özkan İ. The relationship between self-esteem and academic achievement in young people. Proceedings of the National Congress of Psychiatry and Neurological Sciences.

[53] Rubin K.H., Coplan R.J., Bowker J.C. (2009). Social withdrawal in childhood. Annu. Rev. Psychol..

[54] Ahmad Z.R., Bano N., Ahmad R., Khanam S.J. (2013). Social anxiety in adolescents: Does self-esteem matter?. Asian J. Soc. Sci. Humanit..

[55] La Greca A.M., Lopez N. (1998). Social anxiety among adolescents: Linkages with peer relations and friendships. J. Abnorm. Child Psychol..

